# A drug-discovery-oriented non-invasive protocol for protein crystal cryoprotection by dehydration, with application for crystallization screening

**DOI:** 10.1107/S1600576722002382

**Published:** 2022-04-02

**Authors:** Dom Bellini

**Affiliations:** a MRC Laboratory of Molecular Biology, Francis Crick Avenue, Cambridge, Cambridgeshire CB2 0QH, United Kingdom

**Keywords:** cryoprotection, high throughput, dehydration, crystals, proteins

## Abstract

In X-ray macromolecular crystallography, cryoprotection of crystals mounted on harvesting loops is achieved when the water in the sample solvent transitions to vitreous ice before crystalline ice forms. This is achieved by rapid cooling in liquid nitro­gen or propane. Protocols for protein crystal cryoprotection are based on either increasing the environmental pressure or reducing the water fraction in the solvent. This study presents a new protocol for cryoprotecting crystals. It is based on vapour diffusion dehydration of the crystal drop to reduce the water fraction in the solvent by adding a highly concentrated salt solution, 13 *M* potassium formate (KF13), directly to the reservoir. Several salt solutions were screened to identify KF13 as optimal. Cryoprotection using the KF13 protocol is non-invasive to the crystal, high throughput and easy to implement, can benefit diffraction resolution and ligand binding, and is very useful in cases with high redundancy such as drug-discovery projects which use very large compound or fragment libraries. An application of KF13 to discover new crystal hits from clear drops of equilibrated crystallization screening plates is also shown.

## Abstract 2

A one-step dehydration-based protein crystal cryoprotection protocol is presented. It is suitable for both low- and high-throughput projects, and may also benefit ligand occupancy and diffraction resolution. This same procedure can also be applied to discover new crystal hits from clear drops of already vapour diffusion equilibrated crystallization screening plates.

## 1. Introduction

In macromolecular X-ray crystallography, it is important to perform data collection at cryogenic temperatures (usually around 100 K) where crystal radiation damage is significantly slower, especially at high-intensity synchrotron radiation sources (Kmetko *et al.*, 2006 ▸; Owen *et al.*, 2006 ▸). Cryo­protection of loop-harvested crystals is intended to achieve water transition to vitreous ice before crystalline ice formation upon flash cooling in liquid nitro­gen. Crystalline ice formation should be avoided for a number of reasons. First, it compromises diffraction quality by destabilizing the crystal structure due to its volume expansion compared with liquid water (Haas & Rossmann, 1970 ▸; Juers & Matthews, 2001 ▸, 2004 ▸; Kriminski *et al.*, 2002 ▸; Low *et al.*, 1966 ▸). These effects cause disorder and/or non-isomorphism. Second, it causes large variations in the background counts of diffraction images due to X-ray diffraction by cubic and hexagonal ice at specific Bragg angles (known as ‘ice rings’) (Burkhardt *et al.*, 2012 ▸; Fuentes-Landete *et al.*, 2015 ▸; Parkhurst *et al.*, 2017 ▸; Thorn *et al.*, 2017 ▸).

There are two main strategies to cryoprotect macromolecular crystals, which aim either (i) to increase the environmental pressure (Burkhardt *et al.*, 2012 ▸; Kim *et al.*, 2005 ▸; Thomanek *et al.*, 1973 ▸) or (ii) to reduce the solvent fraction to below the glass transition phase of water (Pflugrath, 2015 ▸). Reduction of solvent fraction can be achieved by either (i) soaking crystals in cryosolutions enriched with cryoprotective agents such as sugars, salts, poly­ethylene glycols, glycerol and various others (Bujacz *et al.*, 2010 ▸; Gulick *et al.*, 2002 ▸; Holyoak *et al.*, 2003 ▸; Hope, 1988 ▸; Marshall *et al.*, 2012 ▸; Pemberton *et al.*, 2012 ▸; Rubinson *et al.*, 2000 ▸; Vera & Stura, 2014 ▸; Kwong & Liu, 1999 ▸) or (ii) dehydration. While in some cases an adequate cryoprotective agent is already present in the crystallization buffer, very often an additional step needs to be performed where the crystal is transferred to a cryoprotected solution; this process can be laborious and damaging to the crystals due to handling and osmotic stress, respectively. Crystal handling is avoided in procedures that make use of acoustic nanodroplet ejectors (*e.g.* the Echo acoustic liquid handler from Labcyte Inc.) for precise placement of cryoprotective agents directly within the crystallization drop but away from the crystals and towards the drop edges to allow gradual gentle diffusion; however, the setup of such a pipeline is neither straightforward nor inexpensive, including the requirement for crystal plate imaging facilities (Collins *et al.*, 2017 ▸). In addition, a low-throughput but efficient protocol has been reported for crystal cryoprotection using vapour diffusion of volatile alcohols (Farley & Juers, 2014 ▸). Dehydration studies of macromolecular crystals have instead usually been aimed at improving data resolution rather than cryoprotection (Abergel, 2004 ▸; Esnouf *et al.*, 1998 ▸; Heras *et al.*, 2003 ▸; Kiefersauer *et al.*, 2000 ▸). However, two of these studies have also reported that some crystals, de­hydrated either using a humidity control device (Sanchez-Weatherby *et al.*, 2009 ▸; Bowler *et al.*, 2015 ▸) or by replacing the reservoir in the crystallization plate with NaCl solutions (Douangamath *et al.*, 2013 ▸), no longer required being soaked in cryosolutions to prevent crystalline ice formation during flash cooling.

Besides the main two strategies discussed above, there is a third option which relies on removing all the liquid surrounding the crystal before flash freezing, with the crystal structure itself acting as the cryoprotectant; however, this method is limited to crystals with solvent channels smaller than about 40 Å, otherwise formation of internal ice cannot be avoided in the absence of cryoprotecting conditions (Pellegrini *et al.*, 2011 ▸; Kitago *et al.*, 2010 ▸; Thorne *et al.*, 2003 ▸).

In this study, a new cryoprotection protocol is described in which a solution of 13 *M* potassium formate (KF13) is added in a single step directly to the plate reservoir. This dehydrates the crystal drop overnight by vapour diffusion, thereby cryoprotecting the crystal. This method has successfully cryo­protected six different crystal systems, which were grown in conditions containing different salts and poly­ethylene glycols. The amount of KF13 added to achieve cryoprotection, without over-dehydrating the crystal at the expense of diffraction quality, depends on both the components of the crystallization solution and the crystal solvent content. It is shown to vary between 4 and 20% of the final volume (reservoir plus KF13).

This work also shows that adding KF13 to the reservoir of previously equilibrated crystallization screening plates can promote, through further dehydration by vapour diffusion, the formation of new crystals from ‘idled’ clear drops. This approach provides a new high-throughput protocol to recycle unsuccessful crystallization screening conditions. Clear drops usually account for around 50% of all conditions in a crystallization screening experiment.

## 2. Materials and methods

### 2.1. Chemicals and protein crystals

Precipitants, such as polyethyl­ene glycols and salts, buffers, and chemicals to prepare dehydrating solutions such as potassium formate were all purchased from Sigma. The commercially available proteins *Thaumatococcus daniellii* (an African plant) thaumatin, *Gallus gallus* (hen egg white) lysozyme and *Canavalia ensiformis* (jack bean) concanavalin A were also purchased from Sigma. Crystals of *Staphylococcus aureus* FtsA filaments, *Homo sapiens* hetero-pentamer Cenp-OPQUR complex and glutamate receptor ligand-binding domain in complex with agonist (GluLBD) were kindly donated by MRC Laboratory of Molecular Biology (LMB) researchers Danguole Ciziene, Stan Yatskevich and Christina Heroven, respectively. Crystallization sitting drops were prepared on a Mosquito nanolitre liquid handler (STP Labtech) at 293 K by dispensing equal volumes (200 nl) of protein and reservoir solutions. For the study of cryo­protection by dehydration, crystals of lysozyme, concanavalin A and thaumatin were prepared at various precipitant concentrations, both to study the direct effects of precipitant concentration on cryoprotection and to produce crystals of different sizes, since crystal size can also affect cryo­protection; larger crystals are known to require a higher concentration and/or longer soaking time of cryoprotectant, since the cooling rate in liquid nitro­gen may not be fast enough for the deeper areas in the crystal to avoid formation of ordered crystalline ice (Schall *et al.*, 2005 ▸). To differentiate between samples of the same protein crystallized at different precipitant concentrations, the following acronyms are used throughout this article: (i) lyso07, lyso08 and lyso12 describe lysozyme crystals grown in 0.7, 0.8 and 1.2 *M* NaCl, respectively; (ii) conc11, conc13 and conc14 represent concanavalin A crystals grown in 11, 13 and 14% PEG 6K, respectively; and (iii) thau06 and thau10 describe thaumatin crystals grown in 0.6 and 1.0 *M* NaK tartrate, respectively. Detailed lists have been tabulated of the crystallization conditions for the study of cryoprotection by dehydration (Table 1 ▸) and for the promotion of crystal nucleation in already vapour diffusion equilibrated crystal drops (Table S1 in the supporting information).

### 2.2. Dehydration of crystallization drops

Protein crystallization was carried out in 96-well MRC plates (SWISS-CI) containing 80 µl of reservoir solutions. This type of plate accommodates up to 100 µl of reservoir. Required volumes of KF13, ranging from 0–20 µl, were added directly to the reservoir by either (i) removing the sealing tape completely and using a multi-channel pipette before quickly re-sealing the whole plate with three-inch-wide Crystal Clear sealing tape (Hampton Research), in the case of high-throughput experiments such as the study of promoting crystal nucleation in already equilibrated clear drops, or (ii) making a small cross incision in the sealing tape with a blade and using a single-channel pipette, before re-sealing the cut with a small piece of Crystal Clear tape, in the case of low-throughput experiments such as the study of cryoprotection by dehydration. For crystals of FtsA, Cenp-OPQUR and GluLBD, due to the limited amounts of the samples, experiments were performed only once. In contrast, with the commercially available samples, experiments were performed in duplicate (thaumatin) or triplicate (lysozyme and concanavalin A); however, the concentrations of precipitants used in crystallization did vary slightly amongst the replicate plates to produce crystals of different sizes (see previous section and Table 1 ▸).

### 2.3. Theoretical calculations of relative humidity

The relationship between the precipitant solution and equilibrium relative humidity (RH) is described by Raoult’s law for the equilibrium vapour pressure of water above a solution (Wheeler *et al.*, 2012 ▸). The concentration of buffers, additives and detergents used will have a negligible effect on the RH in equilibrium with the mother liquor, and the RH is dominated by the primary precipitant. The theoretical RH values for the solutions used in this study were calculated using the applet at https://www.esrf.fr/UsersAndScience/Experiments/MX/How_to_use_our_beamlines/forms (Wheeler *et al.*, 2012 ▸; Bowler *et al.*, 2015 ▸, 2017 ▸).

### 2.4. Diffraction data collection

Single diffraction images were acquired on beamline I24 (Diamond Light Source) and the home source (FrE+ SuperBright, Rigaku) for crystals of Cenp-OPQUR and GluLBD, respectively. Complete data sets were collected for crystals of FtsA on beamline I24 and of lysozyme, concanavalin A and thaumatin on I04 (Diamond Light Source). All diffraction data were collected at 100 K and autoprocessed with *Xia2 DIALS* (Winter *et al.*, 2018 ▸).

## 3. Results and discussion

### 3.1. KF13 is an ideal solution for crystal drop dehydration by vapour diffusion

**Figure 1:**
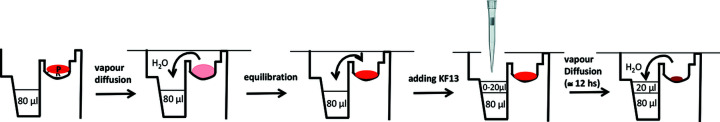
Schematic drawing of the procedures involved in crystal drop dehydration using the KF13 protocol.

All crystallization plates used in this study are 96-well MRC plates. These allow a maximum reservoir volume of 100 µl. Ready-to-use crystallization screening plates prepared at the LMB crystallization facility contain 80 µl of reservoir in each well (significantly lower volumes are not ideal for storing at 283 K due to evaporation issues), allowing the addition of a maximum of 20 µl of extra solution. To achieve further dehydration of equilibrated crystal drops via vapour diffusion by adding some 0–20 µl solution to the reservoir, the overall vapour pressure of water for 80 µl of reservoir plus *x* µl of highly concentrated solution (where *x* equals 0–20) must be lower than that for the original 80 µl reservoir (since the vapour pressure of an equilibrated crystal drop is approximately the same as that of the reservoir). Therefore, to counteract the diluting effect that adding extra solution has on the original reservoir, and to prevent rehydration rather than dehydration, the mole fraction of water of the added solution must be very low (since the reservoir already contains high concentrations of precipitants). A schematic drawing of this process is shown in Fig. 1 ▸.

**Figure 2:**
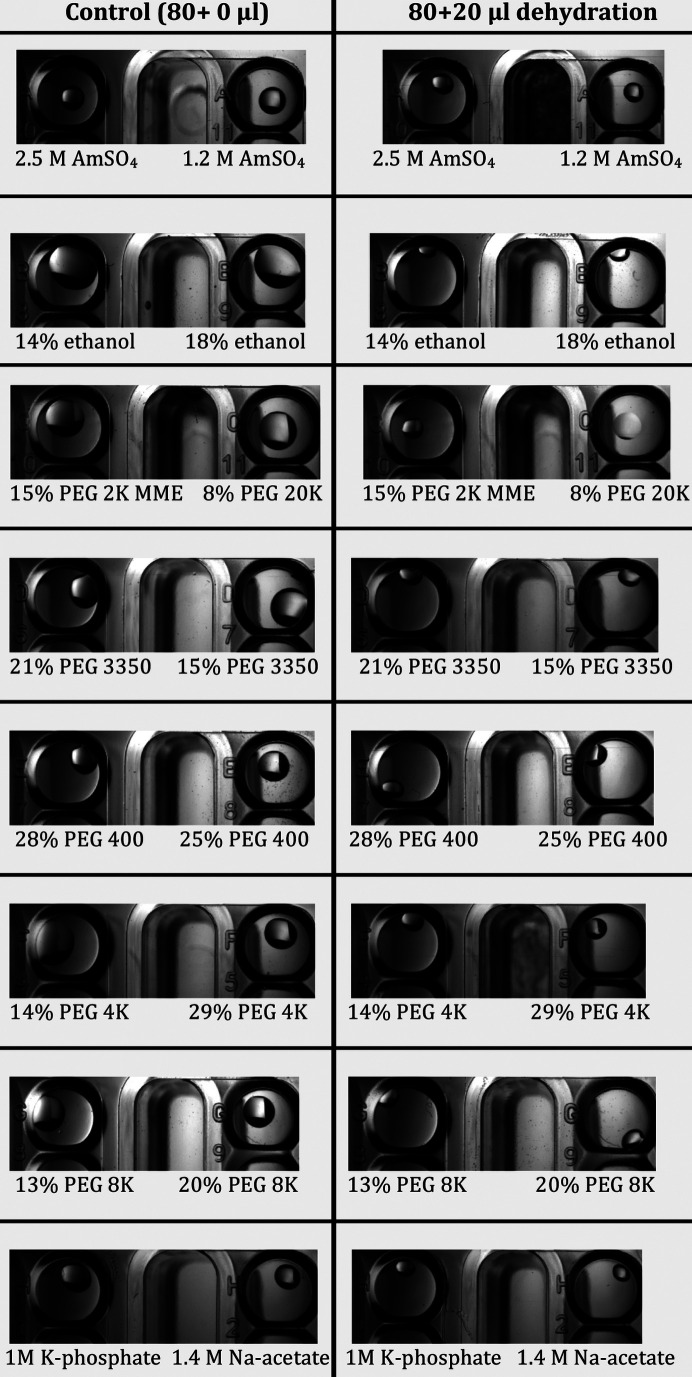
Dehydrating effects on crystallization drops from significantly different conditions after 24 h from adding KF13 to the reservoirs. Initial drop volume was 200 nl of reservoir plus 200 nl of a protein-less solution containing 500 m*M* NaCl. The conditions are from a crystallization plate from our facility and contain 2.5 *M* ammonium sulfate, 0.2 *M* NaCl and 0.1 *M* MES pH 5.6 (A10); 1.2 *M* ammonium sulfate and 0.1 *M* MES pH 5.9 (A11); 14% ethanol and 0.1 *M* ADA pH 6 (B8); 18% ethanol and 0.1 *M* bis-tris propane pH 7.1 (B9); 15% PEG 2K (*w*/*v*) and 0.1 *M* bis-tris propane pH 6.9 (C10); 8% PEG 20K, 8% PEG 2K (*w*/*v*), 0.25 *M* KBr and 0.1 *M* sodium acetate pH 4.5 (C11); 21% PEG 3350, 0.15 *M* NaCl and 0.1 *M* MES pH 6 (D6); 15% PEG 3350 and 0.1 *M* MES pH 6.2 (D7); 28% PEG 400, 0.2 *M* NaCl and 0.1 *M* MOPS pH 6.5 (E7); 25% PEG 400, 4.5% ethanol, 1.5 m*M* MgCl_2_ and 0.07 *M* MES pH 6.6 (E8); 14% PEG 4K, 6% MPD and 0.1 *M* sodium potassium phosphate pH 6.2 (F4); 29% PEG 4K, 0.1 *M* ammonium sulfate, 0.1 *M* magnesium acetate and 0.1 *M* sodium citrate pH 6.5 (F5); 13% PEG 8K, 0.09 *M* ammonium sulfate and 0.05 sodium cacodylate pH 6.5 (G8); 20% PEG 8K, 0.2 *M* magnesium acetate and 0.1 *M* MES pH 6.5 (G9); 1 *M* potassium phosphate monobasic, 3% iso­propanol and 0.1 *M* sodium cacodylate pH 6.5 (H1); 1.4 *M* sodium acetate and 0.1 *M* sodium cacodylate pH 6.5 (H2).

A number of potential candidates to generate very highly concentrated solutions with low vapour pressure of water were tested (Table 2 ▸). Interestingly, it was not possible to achieve the reported maximum solubility at room temperature of most of the tested substances. Thus, the tests were carried out at the maximum achievable solubility without heating the solutions to reach higher concentrations (in this way the solution is more stable and can be stored indefinitely at room temperature without the problem of the solute precipitating if there is a reduction in temperature) (Table 2 ▸). Solutions of caesium acetate and fructose were excluded due to their expense and being too viscous for pipetting, respectively (Table 2 ▸). NaCl, K acetate, CsCl and Cs phosphate failed to dehydrate a large number of crystallization drops whose reservoirs possessed an RH above the solution of 93% or less, whereas KNO_2_ was excluded because it reacted with solutions containing (NH_4_)_2_SO_4_, releasing nitro­gen gas [2KNO_2_ + (NH_4_)_2_SO_4_ → 2N_2_ + K_2_SO_4_ + 4H_2_O] as judged by the bubbling reservoir, which caused the sealing tape to open up in some places due to the build-up of pressure in the well (Table 2 ▸). Solutions of 10 *M* Li iodide and 13 *M* K formate (KF13) proved to be equally efficient in causing crystal drop dehydration in all crystallization conditions tested (Table 2 ▸). However, KF13 was chosen as the preferred solution because it is ten times less expensive than Li iodide. Fig. 2 ▸ shows how adding 20 µl of KF13 to different 80 µl reservoirs containing different precipitants at different concentrations significantly dehydrated all crystal drops.

### 3.2. Cryoprotection by KF13 dehydration

**Figure 3:**
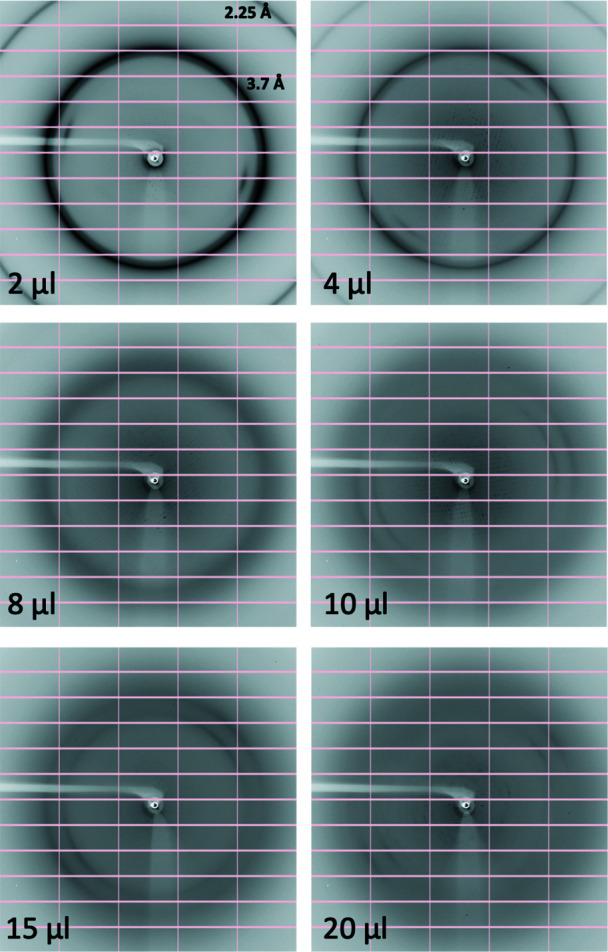
Diffraction images from crystals of FtsA filaments dehydrated by adding different amounts of KF13 to the reservoir. The volume of KF13 that was used and the diffraction resolution of the ice rings are shown.

Tests were conducted using six different protein samples, namely FtsA, Cenp-OPQUR, GluLBD, lysozyme, concanavalin A and thaumatin (Table 1 ▸). Crystals of different sizes were produced for lysozyme, concanavalin A and thaumatin by varying the precipitant concentrations (Table 1 ▸ and Fig. S1). Crystal drops were dehydrated by adding 2, 4, 6, 8, 10, 15 or 20 µl of KF13 directly to the reservoirs (80 µl) and leaving them to equilibrate for 12–24 h. Crystals were then harvested in nylon loops and flash frozen in liquid nitro­gen. Diffraction images highlighting the transition from ice to glass, as judged by the disappearance of ice rings, relative to the amounts of KF13 added to the reservoirs are shown in Figs. 3 ▸–8 ▸ ▸ ▸ ▸ ▸ for each of the six crystal forms, respectively. Table 3 ▸ summarizes these results with an approximate value for the minimum amount of KF13 needed to achieve cryoprotection in relation to the type and amount of precipitant, and the solvent content of the crystals (this value is reported since crystals with high solvent content are more prone to the formation of internal ice during flash freezing, as a high solvent content is often an indication of the presence of a large solvent channel). Despite the crystal solvent content being as high as 72%, crystals from drops containing polyethyl­ene glycols (ranging from 8–20%) only required 4–10 µl of KF13 to achieve cryoprotection. In the case of drops containing salts as precipitants, the amounts of KF13 required were markedly higher, being in the range of 15–20 µl, despite solvent contents as low as 32%. This is in agreement with the fact that the concentrations of polyethyl­ene glycols in crystallization solutions are usually closer to those required to act as cryoprotectants than in the case of salts (Berejnov *et al.*, 2006 ▸). As shown in Table 3 ▸, the variation in the size of the lysozyme and thaumatin crystals (Table 1 ▸ and Fig. S1) did not appear to have any effect on cryoprotection. Most important is the fact that this protocol has achieved cryoprotection even in cases where the precipitant is a very poor cryoprotectant, such as NaCl (Berejnov *et al.*, 2006 ▸), suggesting that this KF13-based approach can be applied to crystals grown in most, if not all, crystallization conditions currently in use in macromolecular crystallography. In this study the diameters of the harvesting nylon loops were chosen to be an approximate match to the size of the crystal, and the X-ray beam cross section was smaller than the crystal size (Fig. S1).

**Figure 4:**
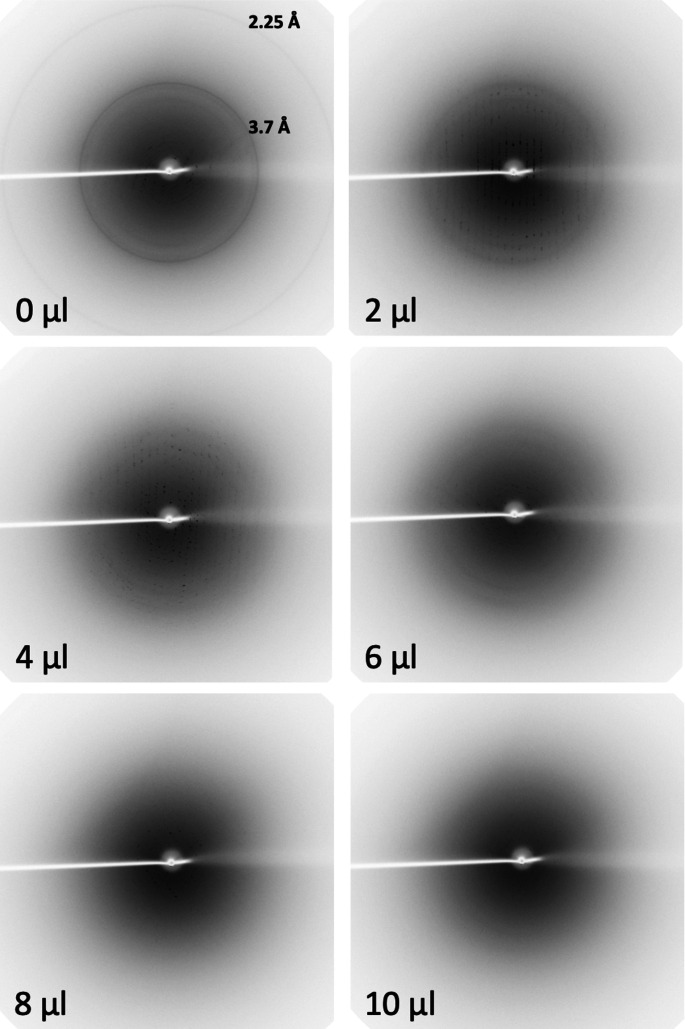
Diffraction images from crystals of GluLBD in complex with agonist dehydrated by adding different amounts of KF13 to the reservoir. The volume of KF13 that was used and the diffraction resolution of the ice rings are shown.

**Figure 9:**
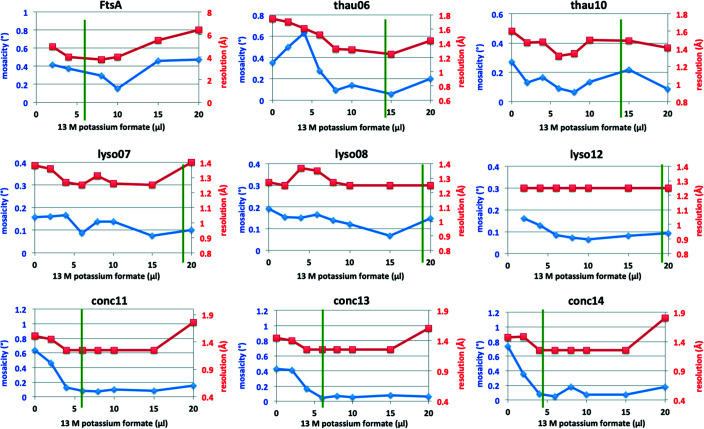
Correlation between amounts of KF13 used for crystal drop dehydration and both mosaicity and diffraction resolution of data sets collected for different crystal samples. The green line indicates the minimum value of KF13 volume that caused the ice rings to disappear.

Complete data sets were collected for FtsA, lysozyme, concanavalin A and thaumatin crystals. In all cases, diffraction data processing showed a strong correlation between the disappearance of the ice rings, lower mosaicity and higher resolution (Fig. 9 ▸), as well as the order in the crystals as judged by the average Wilson *B* factor (Fig. S2). Continuing to increase the amount of KF13 past the point of ice ring disappearance eventually caused an increased mosaicity and reduced resolution [except in the case of lysozyme crystals, where cryoprotection was only reached when adding the maximum amount of KF13 (20 µl) allowed by the well size without the need to remove any of the 80 µl of reservoir]. This also appeared to be true in the case of the single-image data collections from the GluLBD and Cenp-OPQUR samples, as judged from the deterioration in resolution in the diffraction images of crystals from drops dehydrated with KF13 volumes above the optimal 4 and 10 µl, respectively (Figs. 4 ▸ and 5 ▸). The overall amounts of KF13 that can be added to achieve optimal cryoprotection without degrading the diffraction resolution can vary significantly depending on the sample, ranging from a narrow 8–10 µl interval for FtsA crystals to a wider 5–15 µl for concanavalin A crystals (Fig. 9 ▸).

As expected, crystal drop dehydration induced a shrinkage of the unit cell of the crystals that is proportional to the amount of KF13 added to the reservoir, except in the case of thaumatin where no contraction of the unit cell was observed, perhaps suggesting a very rigid structure (Figs. S3 and S4). All other samples underwent an anisotropic contraction of the unit cell, except concanavalin A crystals, where all three unit-cell axes shrank (Figs. S3 and S4). As reported in the *Introduction* [Sec sec1], unit-cell shrinkage and the consequent reduction in the solvent content of crystals by dehydration has been exploited in a number of cases to improve crystal order, and thus the diffraction resolution, especially with crystals of membrane proteins crystallized with detergents. Therefore, cryoprotection by KF13 dehydration may also lead to improved crystal order and better diffraction resolution compared with other cryoprotection techniques that do not cause the unit cell to shrink. Since unit-cell shrinkage is shown to be proportional to the added volume of KF13, the overall amount of KF13 required to achieve full cryoprotection (*e.g.* 6 µl) could be added gradually (*e.g.* 2 µl every 24 h), thereby achieving a more gradual and gentler contraction of the unit cell and optimal diffraction resolution in certain cases.

The KF13 cryoprotection method is ideal for crystallographic drug discovery projects for a number of reasons.

(i) In standard cryoprotection protocols the crystal is transferred to a drop containing a cryoprotective agent, which ideally should also contain the ligand of interest; however, it can be very labour intensive to prepare all the different cryoprotectant solutions, each containing a unique ligand (or even cocktails of ligands) from a large library for each different ligand–complex crystal. This is unnecessary with the KF13 method since the crystallization solution remains untouched.

(ii) Since KF13 cryoprotection is achieved by dehydration, the concentration of the ligand in the drop will rise, causing the binding occupancies to increase and thus improving the probability of observing the ligand in the electron-density map. This latter consideration is also true in the case of *ab initio* structure determination experiments involving metal atom soaks.

(iii) While the KF13 method works well in low-throughput experiments, it is also ideal for very large projects such as screening large libraries of compounds or fragments. In these cases, high throughput can be achieved by adding KF13 either manually using multi-channel pipettes or via an automated pipeline using liquid-handling robots.

The crystal drop dehydration method of replacing the reservoir with an NaCl solution (Douangamath *et al.*, 2013 ▸) was developed with the aim of rapidly screening the effects of dehydration on the diffraction resolution of crystals *in situ*. The KF13 protocol is also perfectly suited for such a purpose, with the advantage that KF13 is added in a single step without necessitating prior removal of the reservoir.

### 3.3. KF13 can aid crystal hit discovery by dehydrating clear drops in pre-equilibrated screening plates

Standard protocols for the identification of new crystal hits consist of screening more than one or two thousand different crystallization conditions, normally using 96-well plates. An important parameter in this process is the choice of protein concentration, which, as a rule of thumb, is taken as the concentration that produces around 50% of clear drops (intended as no observable protein precipitation) immediately after setting up the plates. This means that in any given crystal hit screening experiment, even after achieving drop equilibration by vapour diffusion, there are usually a high percentage of drops that are left clear, with neither crystals nor precipitation. There are a number of reasons why drops remain clear. One is that the concentration of protein or precipitant, or both, is not high enough to reach supersaturation and consequently nucleation. The growth of crystals from clear drops weeks, months or even years after the plates had been set up is likely to be due either to water evaporation from the reservoir through a small leak in the sealing tape (or greased cover slip) and/or to proteolysis. KF13 dehydration can be employed to mimic, while speeding up, this slow evaporation process, thus allowing the discovery of new crystal hits in screening plates that had already reached vapour diffusion equilibration. To investigate this possibility, 96-well crystallization plates for lysozyme and thaumatin were prepared where some of the drops contained too low a concentration of either protein or precipitant (or both) to reach supersaturation. After 10 days, when crystal growth had not been observed for several days, fixed amounts of KF13 (3 µl) were gradually added to the reservoirs every 3 days using a multi-channel pipette. Table 4 ▸ summarizes how increasing amounts of KF13 were required for crystal growth in drops with decreasing amounts of protein or precipitant (or both). Notably, the optimized crystallization conditions used to prepare lysozyme and thaumatin crystals for the KF13 cryoprotection studies discussed above contained a minimum of 0.7 *M* NaCl and 0.6 *M* NaK tartrate, respectively, as precipitants; drop dehydration via KF13 of pre-equilibrated crystallization plates produced crystal hits in drops containing as little as 0.1 *M* NaCl and 0.05 *M* NaK tartrate (lower precipitant concentrations than these were not tested) (Table 4 ▸). As a negative control, the sealing tape was removed from duplicate plates, which were then left open for the same length of time as was required to add KF13 and reseal the original plates. No further crystal growth was observed in the control plates beyond that observed within the first 10 days of vapour diffusion equilibration. This proves that the new crystal hits were indeed the result of KF13 drop dehydration, rather than an effect of water evaporation due to the removal of the sealing tape for about 30 s (which is roughly the time required to add KF13 to all the reservoirs in a 96-well plate using a multi-channel pipette).

Note that both the lysozyme and the thaumatin samples used in the above experiments were prepared by dissolving lyophilized protein into simple water. This is in contrast to the typical approach whereby protein samples used in crystallization contain salts, buffers and perhaps other chemicals (*e.g.* detergents in the case of membrane proteins), whose concentrations will also increase upon drop dehydration, with unpredictable effects on nucleation. Therefore, the procedure of KF13 dehydration of clear drops is different from that of setting up new crystallization plates using a protein sample of higher concentration than the one used in the original plates.

Another important aspect that needed to be assessed was whether KF13 dehydration of crystallization screening drops might cause the appearance of a high number of false positives due to the crystallization of different salts present in most of the crystallization conditions. To investigate this, the two popular Index (Qiagen) and JCSG+ (Hampton) sparse-matrix crystallization screens were set up using a protein-less sample containing only 10 m*M* Tris–HCl pH 8 and 300 m*M* NaCl (200 + 200 nl sitting drops and 80 µl of reservoir); the eight 96-well plates (four for each screen) were left to reach equilibrium by vapour diffusion for one week. Subsequently, these plates were unsealed and 5, 10, 15 and 20 µl of KF13 were quickly added to the reservoirs of each screen, respectively, before resealing the plates. Inspection over the next 7 days showed that none of the drops contained any crystals, either from salt or from other compounds present in the many different drop conditions. This proves that KF13 dehydration is unlikely to cause an issue with false positives in hit screening experiments. It also suggests that proteins play a crucial role in the nucleation and subsequent crystallization of salts, which is in agreement with the fact that salt crystals are commonly obtained from different crystallization conditions depending on the specific protein sample used in the screen.

## 4. Conclusions

This work shows that six different crystal systems were successfully cryoprotected by dehydration of the crystal drop via vapour diffusion. This was achieved by adding a very highly concentrated salt solution directly to the reservoir, without the need to manipulate the crystal drop. Screening of various salt solutions showed that 13 *M* potassium formate (KF13) possesses the ideal vapour pressure of water compared with other tested candidates to create a one-step cryo­protection protocol, since small amounts of KF13 between only 4 and 20% of the final volume (reservoir plus KF13) sufficed to achieve complete cryoprotection in all six crystal forms tested. Being able to limit the added KF13 volume within this range is very important because it means that a reservoir volume of up to 80% of the well capacity can be used in crystallization experiments without the need to remove any of the reservoir to achieve cryoprotection by directly adding KF13 in a single step. Being able to add KF13 without the need to remove any of the reservoir means that the crystallization plate remains unsealed for shorter times (minimizing crystal drop evaporation and consequently giving more control over the experiment outcome and reproducibility) and all the procedures are faster to complete. Although a one-step cryoprotection protocol could also be developed using other salt solutions with a higher vapour pressure of water than KF13, thus having smaller volumes of reservoir in the well and hence allowing the addition of larger volumes of the dehydrating solution, it is preferable to keep the volume of reservoir to a reasonable level (*e.g.* around 60–80 µl in a 96-well MRC plate from SWISS-CI), since too little reservoir in the well can dry out too quickly during storage (*e.g.* pre-filled plates stored at 283 K in our crystallization facility) or even during crystallization screening experiments at room temperature.

As summarized in Table 5 ▸, the KF13 protocol for protein crystal cryoprotection possesses all the qualities of an ideal method, such as being high throughput (unlike protocols for crystal freezing under high pressure or vapour diffusion of volatile alcohols), non-labour intensive (unlike the use of cryoproctective agents in a drug discovery screening experiment, where a different cryosolution would need to be prepared for each different compound/ligand) and non-invasive (no crystal handling during transfer into new cryosolution drops) and not causing drop dilution by adding cryoprotective agents using an acoustic dispenser (in the case of bound ligands this weakens binding affinities).

The method described by Douangamath *et al.* (2013 ▸), which is carried out in two steps, by first completely removing the crystallization reservoir in the well and then replacing this with a solution containing NaCl, scores similarly to the KF13 protocol (Table 5 ▸). This NaCl method may be suitable for small-scale projects where the manual procedure can be carried out by making a small incision in the sealing tape to minimize evaporation while executing the two steps. However, in high-throughput cases where the sealing tape is removed completely to gain easy access to all 96 wells, the crystal drops remain exposed to evaporation for considerably longer than during a single-step procedure. Resealing the plate quickly is key to achieving a slow and controlled dehydration. Moreover, completely replacing the reservoirs with a pure solution of NaCl cannot guarantee such a gentle and slow dehydration rate as if gradually adding small volumes of KF13, with the latter process more likely to benefit the diffraction quality.

Cryoprotection by KF13 is very useful for drug discovery projects that are characterized by high crystal form redundancy (*e.g.* the amount of KF13 to be added to the reservoir to achieve optimal cryoprotection only needs to be established once) and many different ligands (which do not need to be added to KF13 since this is added to the reservoir rather than the drops). Also, dehydration of drops in KF13 cryoprotection can improve the ligand occupancy and/or diffraction resolution.

This study has also shown that crystallization drops in pre-equilibrated plates potentially capable of producing crystals but containing too little of either protein or precipitant (or both) can be pushed into supersaturation, nucleation and crystal growth by exerting further vapour diffusion dehydration by adding KF13 to the reservoir. This aspect has application in crystallization screening experiments (both small scale and high throughput) where ‘idled’ clear drops in plates that have already reached equilibration can be rapidly tested for being undersaturated by adding KF13 to the reservoir.

The supporting information contains figures and tables showing unit-cell contraction upon dehydration by KF13 and crystallization plate setups, respectively, as discussed in the text.

## Supplementary Material

Additional figures and tables. DOI: 10.1107/S1600576722002382/ap5042sup1.pdf

## Figures and Tables

**Figure 5:**
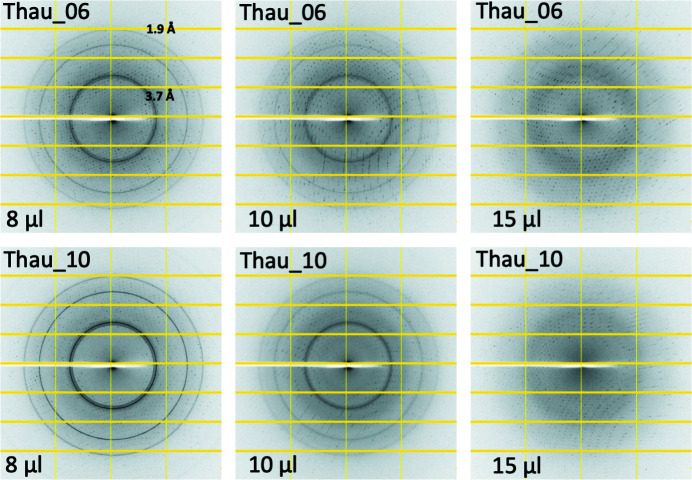
Diffraction images from crystals of the hetero-pentameric complex Cenp-OPQUR dehydrated by adding different amounts of KF13 to the reservoir. The volume of KF13 that was used and the diffraction resolution of the ice rings are shown.

**Figure 6:**
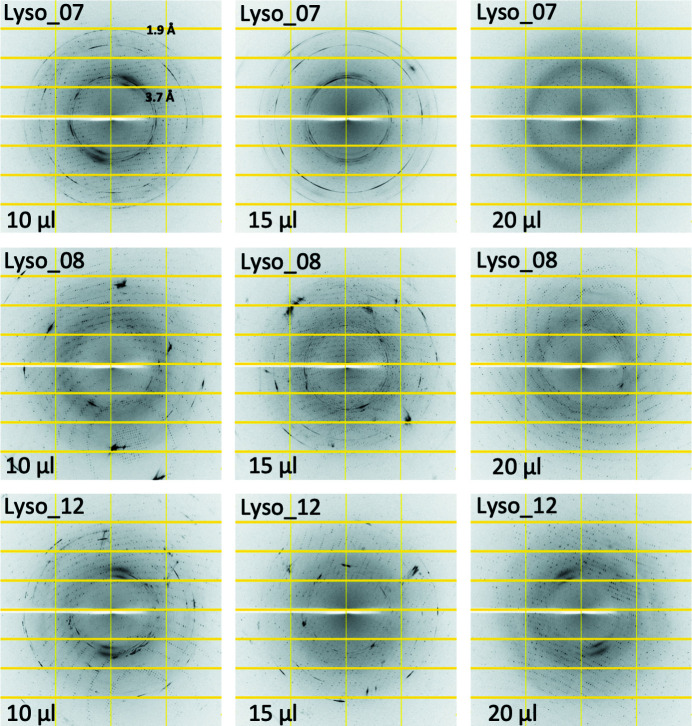
Diffraction images from crystals of lysozyme dehydrated by adding different amounts of KF13 to the reservoir. The volume of KF13 that was used and the diffraction resolution of the ice rings are shown. The number after the sample name indicates the amount of precipitant that was used in crystallization (see *Materials and methods* [Sec sec2] and Table 1 ▸).

**Figure 7:**
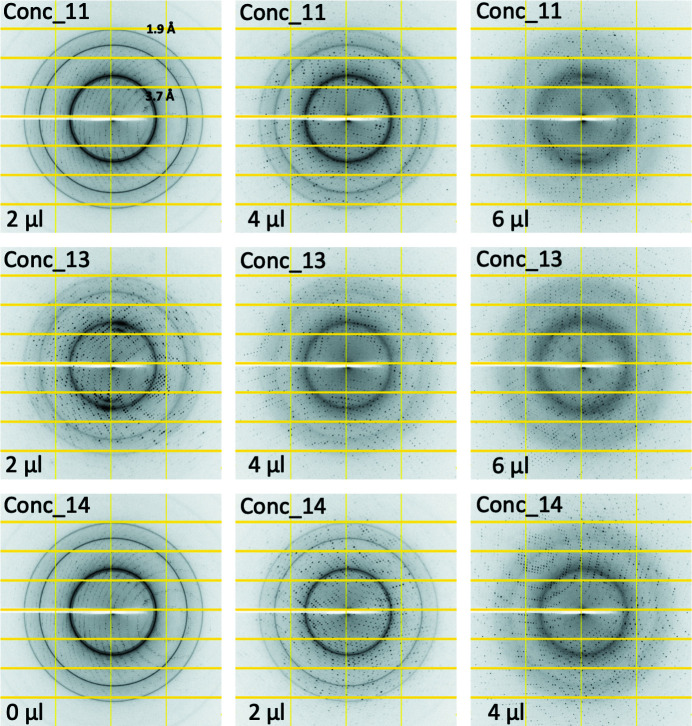
Diffraction images from crystals of concanavalin A dehydrated by adding different amounts of KF13 to the reservoir. The volume of KF13 that was used and the diffraction resolution of the ice rings are shown. The number after the sample name indicates the amount of precipitant that was used in crystallization (see *Materials and methods* [Sec sec2] and Table 1 ▸).

**Figure 8:**
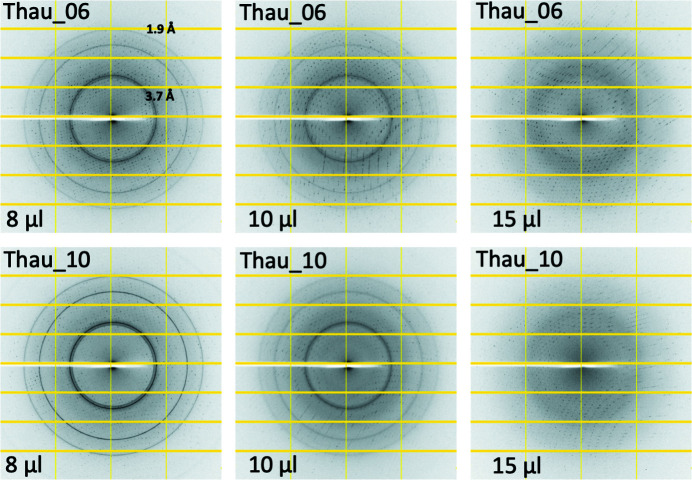
Diffraction images from crystals of thaumatin dehydrated by adding different amounts of KF13 to the reservoir. The volume of KF13 that was used and the diffraction resolution of the ice rings are shown. The number after the sample name indicates the amount of precipitant that was used in crystallization (see *Materials and methods* [Sec sec2] and Table 1 ▸).

**Table 1:** Crystal sizes and forms, crystallization conditions, and X-ray beam sizes used in the study of cryoprotection using the KF13 protocol

Sample	Protein concentration (mg ml^−1^)	Crystal size (µm), shape	X-ray beam size (µm)	Space group	Crystallization conditions	RH of crystallization conditions (%)
FtsA	14	∼300, rod	50 × 50	*I*222	8% PEG 8K, 8% PEG 1K, 200 m*M* Li_2_SO_4_ and 100 m*M* Tris pH 8.5	98.5
Cenp-OPQUR	15	∼400, thin plate	50 × 50	*C*2	15% PEG 2K (*w*/*v*), 40 m*M* Na formate and 200 m*M* bis-tris propane pH 6.9	99
GluLBD	10	∼150, rod	400 × 400 (home source)	*P*2_1_2_1_2_1_	20% PEG 4K, 200 m*M* CaCl_2_ and 100 m*M* Tris pH 8	98
Lyso07	12	∼50, diamond	11 × 5	*P*4_3_2_1_2	0.7 *M* NaCl and 50 m*M* sodium acetate pH 4.5	97.3
Lyso08	40	∼50, diamond	11 × 5	*P*4_3_2_1_2	0.8 *M* NaCl and 50 m*M* sodium acetate pH 4.5	97
Lyso12	40	∼200, diamond	11 × 5	*P*4_3_2_1_2	1.2 *M* NaCl and 50 m*M* sodium acetate pH 4.5	95.7
Conc11	12	∼200, cuboid	11 × 5	*I*222	11% PEG 6K, 5% pentane­diol and 100 m*M* HEPES pH 7.5	99.6
Conc13	12	∼200, cuboid	11 × 5	*I*222	13% PEG 6K, 5% pentane­diol and 100 m*M* HEPES pH 7.5	99.5
Conc14	12	∼200, cuboid	11 × 5	*I*222	14% PEG 6K, 5% pentane­diol and 100 m*M* HEPES pH 7.5	99.5
Thau06	25	∼200, diamond	11 × 5	*P*4_1_2_1_2	0.6 *M* NaK tartrate and 100 m*M* potassium phosphate pH 6.8	96.2
Thau10	25	∼50, diamond	11 × 5	*P*4_1_2_1_2	1.0 *M* NaK tartrate and 100 m*M* potassium phosphate pH 6.8	93.7

**Table 2:** Chemicals tested for the preparation of suitable solutions to be added to crystallization plate reservoirs for drop dehydration by vapour diffusion Predicted RH values could not be calculated for 14 *M* caesium acetate and 8 *M* caesium phosphate as Raoult’s law appears to break down, resulting in a negative value for the former and a value above 100 for the latter.

Chemical	Theoretical maximum concentration (*M*)	Achieved maximum concentration (*M*)	RH for achieved maximum concentration (%)	Performance in dehydration experiments
NaCl	6	6	79.5	Too weak
Fructose	20	8	58.14	Too viscous
K acetate	20	8	63.64	Too weak
CsCl	8	6	77.6	Too weak
Cs acetate	40	14	N/A	Too expensive
Cs_3_ phosphate	10	8	N/A	Too weak
KNO_2_	25	10	60.66	Formation of gas in combination with (NH_4_)_2_SO_4_
Li iodide	10	10	65.12	OK
K formate	30	13	47.77	OK

**Table 3:** Summary of KF13 volumes required to achieve cryoprotection by vapour diffusion dehydration in crystals grown from different conditions and with different solvent contents The ΔRH column shows the difference between the RH values of the starting reservoir (Table 1 ▸) and after adding KF13 (this table).

Sample	Precipitant	Crystal solvent content (%) (Matthew’s coefficient)	Minimum volume of KF13 required for cryoprotection (µl)	RH of reservoir after adding KF13 (%)	ΔRH (%)
FtsA	8% PEG 8K, 8% PEG 1K	72 (4.34)	10	93.2	5.3
GluLBD	20% PEG 4K	57 (2.8)	4	95.7	2.3
Cenp-OPQUR	15% PEG 2K (*w*/*v*)	50 (2.48)	8	94.7	4.3
Lyso_07	0.7 *M* NaCl	32 (2)	20	87.7	9.6
Lyso_08	0.8 *M* NaCl	32 (2)	20	87.4	9.6
Lyso_12	1.2 *M* NaCl	32 (2)	20	86.1	9.6
Conc_11	11% PEG 6K	46 (2.2)	6	96.3	3.3
Conc_13	13% PEG 6K	46 (2.2)	6	96.2	3.3
Conc_14	14% PEG 6K	46 (2.2)	5	96.7	2.8
Thau_06	0.6 *M* NaK tartrate	55 (2.8)	15	88.7	7.5
Thau_10	1.0 *M* NaK tartrate	55 (2.8)	15	86.2	7.5

**Table:** Crystallization plates were left to equilibrate for 10 days before 3 µl of KF13 were added at intervals of 3 days; plates were monitored daily after the first 10 days of equilibration without adding any KF13. RH values after adding KF13 can be compared with those of the starting reservoir in Table S1.

96-well plate rows	NaCl (*M*)	Lysozyme (mg ml^−1^)	KF13 required for crystal appearance (µl)	RH after adding KF13 (%)	Time for crystal appearance (days)
*A*	0.8	20	0	97	10
		10	0		10
*B*	0.7	20	0	97.3	10
		10	0		10
*C*	0.6	20	0	97.6	10
		10	0		10
*D*	0.5	20	0	98	10
		10	3	96.3	+1
*E*	0.4	20	3	96.6	+1
		10	3		+1
*F*	0.3	20	3	97	+1
		10	3		+1
*G*	0.2	20	3	97.4	+1
		10	3		+1
*H*	0.1	20	3	97.7	+1
		10	3+3	96.1	+1

**Table:** 

96-well plate rows	NaK tartrate (*M*)	Thaumatin (mg ml^−1^)	KF13 required for crystal appearance (µl)	RH after adding KF13 (%)	Time for crystal appearance (days)
*A*	0.7	12	0	95.5	10
		6	0		10
*B*	0.6	12	0	96.2	10
		6	0		10
*C*	0.5	12	0	96.8	10
		6	0		10
*D*	0.4	12	0	97.4	10
		6	3	95.7	+1
*E*	0.3	12	3	96.3	+1
		6	3		+1
*F*	0.2	12	3	96.8	+1
		6	3		+3
*G*	0.1	12	3	97.4	+3
		6	3+3	95.8	+1
*H*	0.05	12	3+3	96.1	+1
		6	3+3		+1

**Table 5:** Summary of the advantages and disadvantages of the cryoprotection methods currently available in macromolecular crystallography

Cryoprotection strategy	No crystal handling	No osmotic shock	No crystal drop dilution	No preparation of cryosolution with or without ligand(s)	Increase in ligand affinity by dehydration	Possible increase in diffraction resolution by dehydration	Single-step protocol	High throughput
KF13	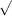	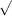	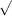	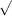	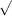	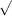	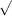	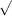
Crystal soaking in cryosolutions	×	×	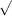	×	×	×	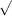	×
Humidity control device	×	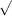	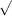	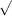	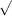	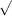	×	×
Vapour diffusion of volatile alcohols	×	×	×	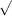	×	×	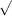	×
Acoustic dispenser	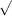	×	×	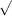	×	×	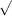	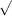
Increase in environmental pressure	×	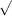	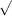	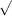	×	×	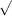	×
Reservoir replacement by NaCl	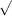	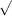	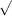	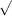	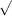	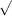	×	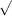 /×
