# Supplementary material for: A drug-discovery-oriented non-invasive protocol for protein crystal cryoprotection by dehydration, with application for crystallization screening
Source: J Appl Crystallogr. 2022 Apr 2;55(Pt 2):370–9. doi: 10.1107/S1600576722002382 (PMC8985602; doi:10.1107/S1600576722002382)
Supplement: Supplementary file 1 [file j-55-00370-sup1.pdf]

JOURNAL OF APPLIED CRYSTALLOGRAPHY

Volume 55 (2022)

Supporting information for article:

A drug discovery-oriented non-invasive protocol for protein crystal cryoprotection by dehydration, with application for crystallization screening

Dom Bellini

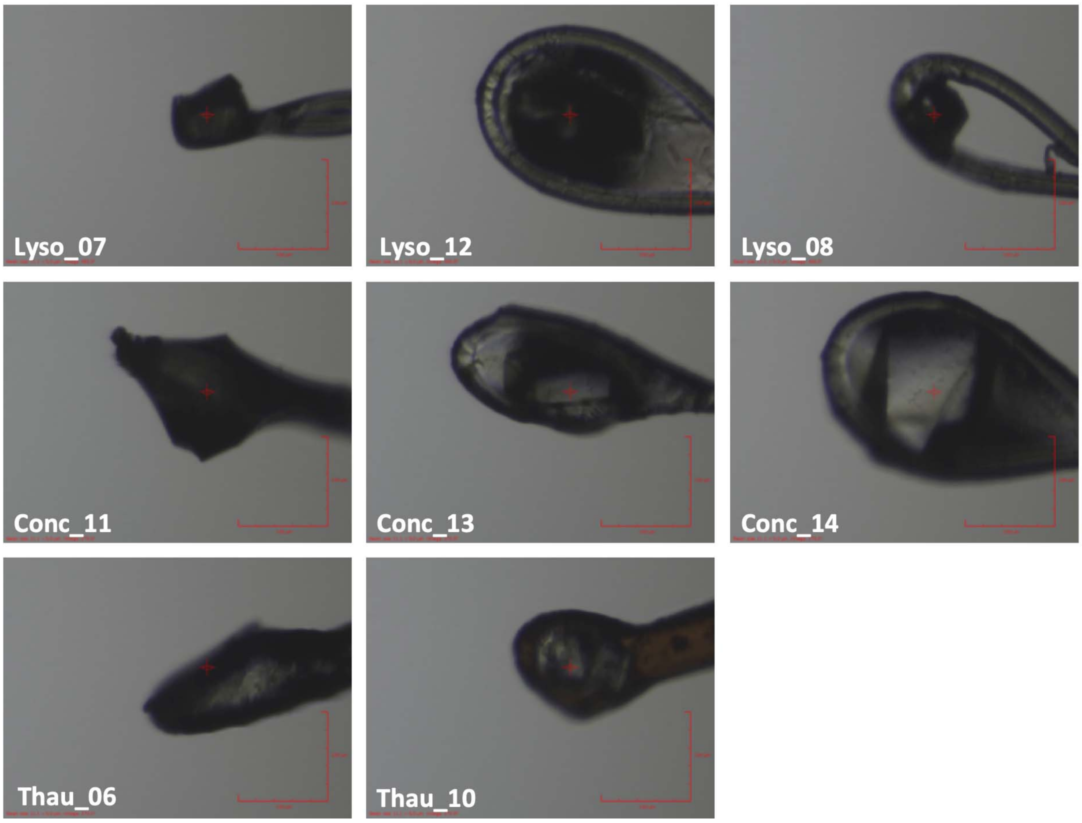

Figure S1. Examples of choices of loop size in comparison to the size of the crystals. Also the X-ray beam size is shown by the red circle with a cross-hair in the middle.

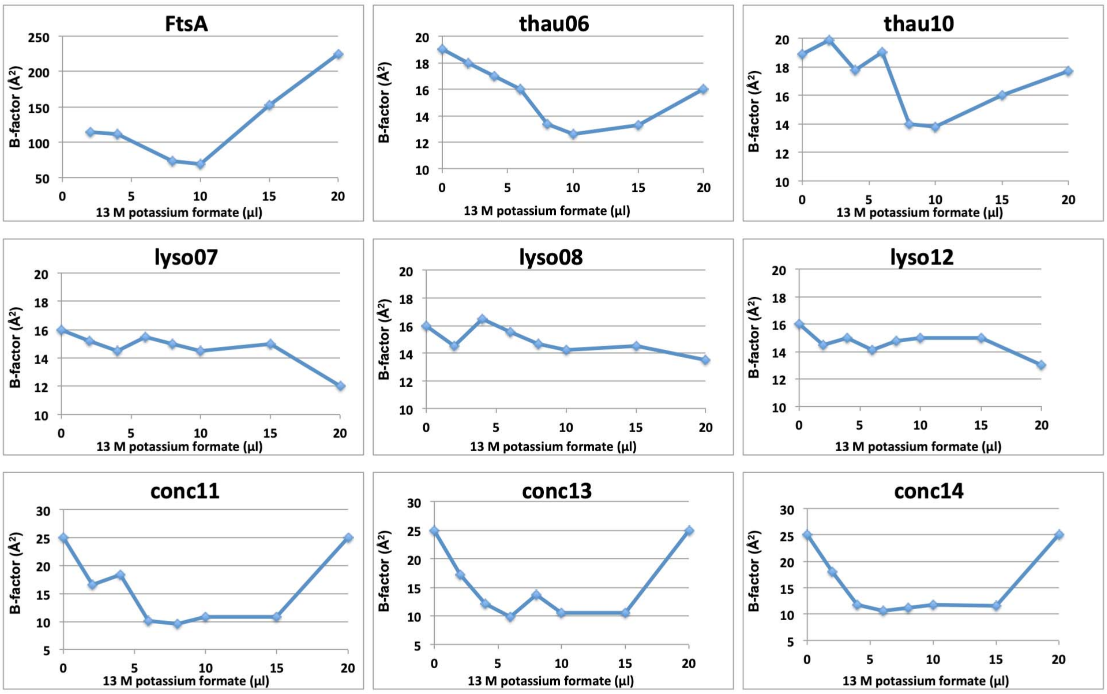

Fig. S2. Correlation between amounts of KF13 used for crystal drop dehydration and average Wilson B-factor.

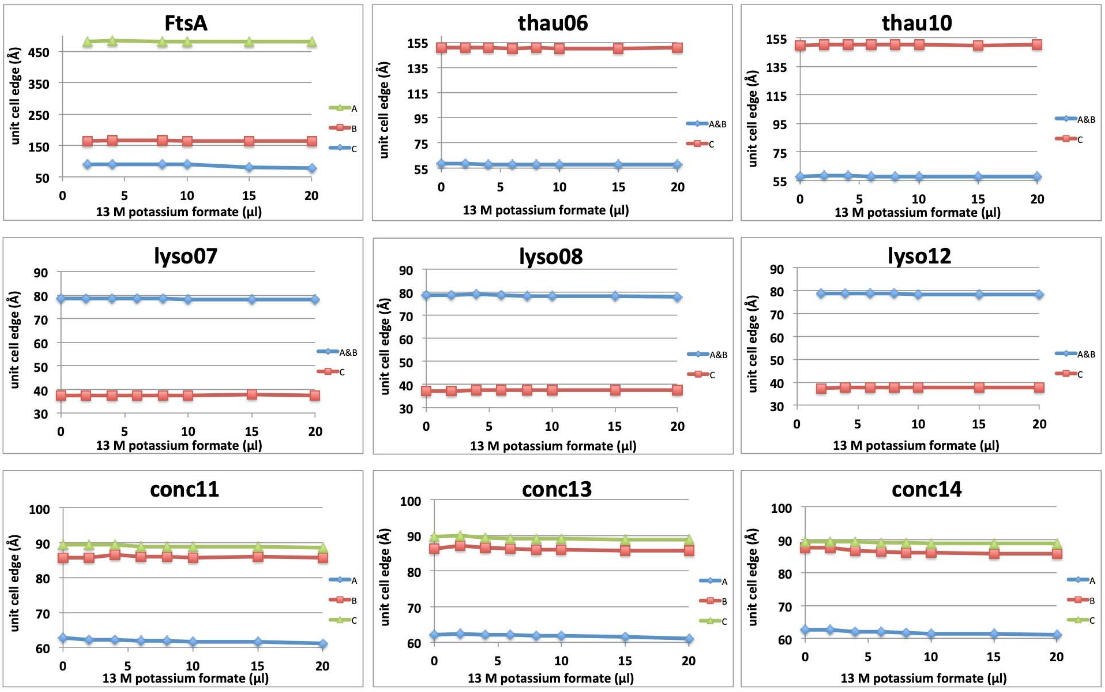

Fig. S3. Correlation between amounts of KF13 used for crystal drop dehydration and unit cell contraction of datasets collected for different crystal samples.

FtsA

KF13 (ul)	a	b	c
2	90.7	164.1	481.70
4	89.7	165.2	482.75
8	88.9	165.2	481.30
10	88.5	164.5	481.60
15	79.3	164.3	480.70
20	77.9	164.3	480.95

Lyso07

KF13 (ul)	a = b	c
0	78.8	37.38
2	78.74	37.46
4	78.45	37.44
6	78.51	37.51
8	78.54	37.54
10	78.25	37.55
15	78.33	37.77
20	78.16	37.40

Lyso08

KF13 (ul)	a = b	c
0	78.77	37.17
2	78.64	37.32
4	79.13	37.69
6	78.67	37.60
8	78.51	37.54
10	78.31	37.58
15	78.29	37.62
20	78.11	37.40

Lyso12

KF13 (ul)	a = b	c
0	unprocessable	unprocessable
2	78.69	37.35
4	78.56	37.55
6	78.56	37.57
8	78.54	37.61
10	78.29	37.55
15	78.29	37.54
20	78.24	37.52

Conc11

KF13 (ul)	a	b	c
0	62.87	85.56	89.51
2	62.17	85.59	89.40
4	62.12	86.57	89.30
6	61.74	86.09	88.94
8	61.87	86.13	88.99
10	61.53	85.76	88.86
15	61.49	85.85	88.82
20	61.12	85.66	88.50

Conc13

KF13 (ul)	a	b	c
0	62.19	86.38	89.54
2	62.26	86.99	89.80
4	62.08	86.5	89.24
6	62.03	86.37	89.21
8	61.77	86.07	89.03
10	61.79	86.11	89.07
15	61.62	85.86	88.90
20	61.05	85.70	88.70

Conc14

KF13 (ul)	a	b	c
0	62.60	87.5	89.50
2	62.60	87.5	89.45
4	62.09	86.52	89.26
6	62.00	86.37	89.10
8	61.68	86.11	89.05
10	61.51	85.91	88.92
15	61.49	85.78	88.87
20	61.04	85.60	88.90

Fig. S4. Tables with values plotted in Fig. S2.

Table S1. Crystallisation conditions in the 96-well plates used to investigate promotion of crystal nucleation in already equilibrated crystals drops. Each table corresponds to a full 96-well plate, each letter corresponds to a row with 12 identical repeats and two drops with different protein concentrations were set up for each condition.

96-well plate rows	Lysozyme (mg/ml)	NaCl (M)	RH (%)
A	20	0.8	97
	10		
B	20	0.7	97.3
	10		
C	20	0.6	97.6
	10		
D	20	0.5	98
	10		
E	20	0.4	98.3
	10		
F	20	0.3	98.7
	10		
G	20	0.2	99.1
	10		
H	20	0.1	99.4
	10		

96-well plate rows	Thaumatin (mg/ml)	NaK tartrate (M)	RH (%)
A	12	0.7	95.5
	6		
B	12	0.6	96.2
	6		
C	12	0.5	96.8
	6		
D	12	0.4	97.4
	6		
E	12	0.3	98
	6		
F	12	0.2	98.5
	6		
G	12	0.1	99.1
	6		
H	12	0.05	99.4
	6		
